# Transcriptome Analysis Reveals Differences in Anthocyanin Accumulation in Cotton (*Gossypium hirsutum* L.) Induced by Red and Blue Light

**DOI:** 10.3389/fpls.2022.788828

**Published:** 2022-03-31

**Authors:** Dongnan Shao, Qian-hao Zhu, Qian Liang, Xuefeng Wang, Yanjun Li, Yuqiang Sun, Xinyu Zhang, Feng Liu, Fei Xue, Jie Sun

**Affiliations:** ^1^Key Laboratory of Oasis Eco-Agriculture, College of Agriculture, Shihezi University, Shihezi, China; ^2^CSIRO Agriculture and Food, Canberra, ACT, Australia; ^3^Plant Genomics and Molecular Improvement of Colored Fiber Laboratory, College of Life Sciences and Medicine, Zhejiang Sci-Tech University, Hangzhou, China

**Keywords:** anthocyanin biosynthesis, light quality, RNA-seq, *GhHY5*, *Gossypium hirsutum* L.

## Abstract

Many factors, including illumination, affect anthocyanin biosynthesis and accumulation in plants. light quality is the key factor affecting the process of photoinduced anthocyanin biosynthesis and accumulation. We observed that the red color of the Upland cotton accession Huiyuan with the *R1* mutation turned to normal green color under light-emitting diodes (LEDs), which inspired us to investigate the effect of red and blue lights on the biosynthesis and accumulation of anthocyanins. We found that both red and blue lights elevated accumulation of anthocyanins. Comparative transcriptomic analyses, including Gene Ontology (GO), Kyoto Encyclopedia of Genes and Genomes (KEGG) and GSEA, revealed that genes differentially expressed under different light conditions were enriched with the pathways of circadian rhythm, phenylpropanoid biosynthesis, anthocyanin biosynthesis, and flavone and flavonol biosynthesis. Not surprisingly, all the major structural genes related to biosynthesis of anthocyanins, including the key regulatory MYB transcription factor (*GhPAP1D*) and anthocyanin transporter (*GhGSTF12*), were induced by red or blue light treatment. However, *LARs* and *MATEs* related to biosynthesis of proanthocyanidins were more significantly up-regulated by red light radiation than by blue light radiation. Vice versa, the accumulation of anthocyanins under red light was not as high as that under blue light. In addition, we demonstrated a potential role of *GhHY5*, a key regulator in plant circadian rhythms, in regulation of anthocyanin accumulation, which could be achieved via interaction with *GhPAP1D*. Together, these results indicate different effect of red and blue lights on biosynthesis and accumulation of anthocyanins and a potential module including *GhHY5* and *GhPAP1D* in regulation of anthocyanin accumulation in cotton. These results also suggest that the substrates responsible the synthesis of anthocyanins under blue light is diverted to biosynthesis of proanthocyanidin under red light.

## Introduction

Anthocyanins, a type of flavonoids, are natural water-soluble pigments that endow most angiosperms with colorful pigment, making their vegetative and reproductive tissues pink, red, purple, or blue (Chen et al., 2019). Anthocyanins are mainly accumulated in vacuoles of plant cells and have various biological significance depending on the tissues or organs in which they are located. For example, anthocyanins accumulated in flowers or fruits enrich their colors and attract pollinators and fruit disseminators (Miller et al., 2011). Anthocyanins in vegetative organs, such as roots, stems and leaves, can protect plants against abiotic and biotic stresses. such as UV, cold/salt/drought stress, diseases and insects, as well as herbivores (Li et al., 2017, 2019; Long et al., 2019; Gong et al., 2020; Kim et al., 2020). Anthocyanins accumulated in seeds are endogenous antioxidants to protect embryo and endosperm, and contribute to seed dormancy (Lepiniec et al., 2006). In addition, anthocyanins are favored by people because of their antioxidant, antimutagenic, cardiovascular and cerebrovascular disease prevention, liver protection and tumor cell inhibition (Tapas et al., 2008). To date, anthocyanin biosynthesis, one branch of the flavonoid biosynthesis pathway, has been almost completely elucidated. Firstly, phenylalanine, as the precursor of anthocyanin biosynthesis, is catalyzed by phenylalanine ammonia lyase (PAL), cinnamate 4-hydroxylase (C4H) and 4-coumarin: Co-A ligase (4CL) to form Coumaric acid coenzyme A. Secondly, one molecule of coumaric acid and three molecules of malonyl-CoA produce colorless anthocyanins through catalysis of enzymes, such as chalcone synthase (CHS), chalcone isomerase (CHI), flavanone 3-hydroxylase (F3H), and dihydroflavonol-4 reductase (DFR). Then, colorless anthocyanins are catalyzed by anthocyanin synthase/leucocyanidin dioxygenase (ANS/LDOX) and uridine diphosphate-glucose: phosphate-glucosyltransferase (UFGT) to form a stably colored anthocyanin (He et al., 2019; Zhang S. et al., 2019). Finally, stable anthocyanins are transported into vacuoles to color the corresponding tissues. Three modes have been proposed for vacuolar transport of anthocyanins. (1) Anthocyanins are targeted to vacuoles by glutathione *S*-transferase (GST) and then recognized and transported across the membranes into vacuoles by the C-type of ABC (ABCC) transporters. (2) Anthocyanins are transported into vacuole by multidrug and toxic compound extrusion (MATE) transporters located on vacuole. This process requires H^+^ concentration gradient generated by H^+^-ATPase proton pump. (3) Anthocyanins are encapsulated by anthocyanin vacuole inclusions (AVIs) and transported into vacuoles by means of membrane fusion (Zhao and Dixon, 2010). Besides the structural genes mentioned above, anthocyanin biosynthesis is also controlled by regulatory genes. In recent decades, studies have confirmed that the expression of the structural genes mentioned above has different degrees of synergy, which is directly controlled by the MBW complex formed by MYB, bHLH, and WDR transcription factors (Xu et al., 2015; Zhang B. et al., 2019). In addition to MBW complex, several transcription factors (TFs) have also been reported to regulate anthocyanin biosynthesis via interaction with the MBW complex or acting on the upstream of MBW complexes, such as DELLA, JAZ, COP1, PIF3, SPL, WRKY, B-box protein and ZIP (Shin et al., 2007; Li et al., 2012; Maier et al., 2013; Binkert et al., 2014; Xie et al., 2016; Qian et al., 2017; An et al., 2018, 2019a,2019b; Bai et al., 2019a,b).

As one of the most important environmental stimulants, light can control the synthesis of anthocyanins by influencing photosynthesis and consequently the synthesis of sugar, phenylalanine and other precursor substances, and by regulating the expression or activity of structural genes and regulatory genes of the anthocyanin biosynthesis pathway (Wei et al., 2017). In the process of photoinduced anthocyanin synthesis, light quality is one of the key factors affecting the synthesis and accumulation of anthocyanins (Zhang et al., 2018; Kim et al., 2020; Samkumar et al., 2021). In addition, the effects of light quality on the expression of genes related to anthocyanin biosynthesis are different among species (Tao et al., 2018; An et al., 2020; Kim et al., 2020; Samkumar et al., 2021). Higher plants employ multiple sensory photoreceptors to coordinate their response to different lights ranging from UV-B to far-red wavelengths. These photoreceptors including phytochrome (PHYA-PHYE) induced by red/far red light, cryptochromes (CRYs) and phototropins (PHOTs) absorbing UV-A/blue light, as well as UV resistance locus 8 (UVR8) sensing UV-B (Jiang et al., 2016; Zhang et al., 2016; Tao et al., 2018; Ma et al., 2019). Downstream of the photoreceptors, the E3 ubiquitin ligase *COP1* (*CONSTITUTIVELY PHOTOMORPHOGENIC1*) is a photomorphogenic inhibitor that acts as a molecular switch during photomorphogenesis and anthocyanin biosynthesis (Li et al., 2012). In the dark, COP1 promotes the degradation of *ELONGATED HYPOCOTYL 5* (*HY5*), a basic leucine zipper (bZIP) transcription factor and a master regulator in light signal transduction pathway that acts downstream of multiple photoreceptors to respond to photomorphogenesis (Xu, 2020). A large number of studies have shown that light with different wavelengths can directly or indirectly regulate the related enzyme genes and transcription factors in the anthocyanin synthesis pathway through the corresponding photoreceptors. In *Anthocyanin fruit* (*Aft*) tomato, UV-B+ blue light improves the anthocyanin concentration by activating the expression of nitrate reductase and then enhancing the genes related to anthocyanin synthesis (Kim et al., 2020). Anthocyanin synthesis under the influence of blue light has also been studied in strawberries and pears. It has been found that blue light irradiation increased the content of anthocyanin in strawberry fruits by increasing the activity of related enzymes in the anthocyanin synthesis pathway (Xu et al., 2014; Zhang et al., 2018). Blue light contributed to anthocyanin biosynthesis through the CRY-COP1-HY5 module in red pear while red light had almost no effect (Tao et al., 2018). It is worth noting that although red light is not the main factor of anthocyanin accumulation, it can still fine tune the anthocyanin synthesis genes through RING-finger type ubiquitin *COP1* or *HY5* (Zoratti et al., 2014). For instance, during strawberry fruit development, red light induced anthocyanin accumulation, although it was lower than that induced by blue light (Zhang et al., 2018).

Cotton (*Gossypium hirsutum* L.), as a crop of *Malvaceae*, has important agricultural and economic value. In particular, cotton fiber is the main source of natural fiber in the textile industry. Among them, Naturally colored cotton (NCC) has attracted much attention as an environment-friendly resource of fiber. A variety of fiber colors has been observed in different cotton species, such as light green, tan, dark brown and red, with brown and green being the dominant in the cultivated cotton (Sun et al., 2021). The molecular basis controlling the biosynthesis and accumulation of pigments in NCC fiber is largely unknown, although previous studies have shown that the production of brown cotton and green cotton is related to proanthocyanidins (or their derivatives) and caffeic acid (or their derivatives), respectively (Lu et al., 2017; Sun et al., 2019). The biosynthesis of proanthocyanidins and caffeic acid is realized by the branch of phenylpropane biosynthesis pathway, which is also the initial pathway of anthocyanin biosynthesis. Among them, biosynthesis of proanthocyanidins and anthocyanins shares most of the structural and regulatory genes. To date, little is known about the mechanism of anthocyanin synthesis in cotton. The research on red leaf cotton is mainly focus on the mutation of anthocyanin synthesis related structural genes and regulatory genes. The MYB transcription factor *GhPAP1A* (*Rs*, Sub-red plant) and *GhPAP1D/GhRLC1* (*R1*, Red plant) play major roles in anthocyanin biosynthesis. Cotton plants with the *R1* mutation exhibit red to purple color in leaves, stems, petals and bolls. The *Rs* mutant shows a lighter red phenotype compared to *R1* (Gao et al., 2013; Li et al., 2019; Liang et al., 2020). It should be noted that the phenotypes of *R1* and *Rs* were determined by 228-bp and 50-bp tandem repeats in the promoter of *GhPAP1D* and *GhPAP1A* (a pair of homoeologs encoding MYB transcription factors), respectively (Gao et al., 2013; Liang et al., 2020). In addition, based on our previous study, *GhGSTF12* is involved in the accumulation of anthocyanins in Upland cotton leaves (Shao et al., 2021).

Considering that red and blue light can affect anthocyanin synthesis and accumulation in other plants and little is known on the topic in cotton. In this study, using Huiyuan, a red leaf Upland cotton cultivar (*Gossypium hirsutum* L.), and comparative transcriptomic analysis, we explored the effect of blue and red light on accumulation of anthocyanins and genes associated with anthocyanin biosynthesis. The red and blue light emitting diodes (LEDs) used in the study give us an opportunity to have a better understanding of the role of light quality in anthocyanin biosynthesis and accumulation in cotton.

## Materials and Methods

### Plant Materials and Light Treatments

XinLuZao 61 (X61, a green leaf cultivar, *Gossypium hirsutum* L.). and Huiyuan (a red leaf cultivar, *Gossypium hirsutum* L.) were used in this study. The plants were grown in the experimental field of Shihezi University (Shihezi, Xinjiang, China).

Before the second true leaf grows, seedlings of the red cotton Huiyuan were grown in a climate chamber at 25°C with a photoperiod of 16 h/8 h-day/night; then the seedlings were divided into three groups, and each group was treated with white light (HW; 15.90 μmol/m^2^/s), white light plus blue light (HB; 450 nm, 20.39 μmol/m^2^/s) or white light plus red light (HR; 630 nm, 15.15 μmol/m^2^/s). When the fourth true leaves are fully expanded, the third true leaves were harvested for RNA extraction, quantitative reverse-transcription PCR (qRT-PCR) and RNA-seq analyses (each treatment has three biological replicates).

*Nicotiana benthamiana* was used in the GUS staining assay and luciferase reporter assay. The plants were grown in a mixture of vermiculite and peat moss (2:1) in a growth chamber at 25°C with a 14/10 h day/night photoperiod.

### Extraction and Measurement of Anthocyanins and Proanthocyanidins

Anthocyanin in cotton hypocotyls, leaves and petals were extracted and quantified as previously described and amended slightly (Wang et al., 2018). Approximately 0.1 g fresh samples were ground to a fine powder in liquid nitrogen, and extracted with 1 mL acidic methanol (1% hydrochloric acid, w/v) at room temperature for 12 h in dark. After centrifugation at 12,000 rpm for 10 min at 4°C, 1 mL of the supernatant was added to 4 mL of acidic methanol. The absorbance of the solution was measured with a U – 5100 UV/VIS spectrophotometer (Shimadzu, Kyoto, Japan) at 530, 620, and 650 nm. The anthocyanin content was calculated with the following formula: *Q* = OD_λ_ /ε × *V*/*m* × 10^6^, OD_λ_ = (A530 − A620) − 0.1 × (A650 − A620). [V (ml): Total volume of extract; m (g): Fresh weight of sample; ε: Molar extinction coefficient of anthocyanins 4.62 × 10^6^].

Proanthocyanidin content was determined using an improved DMAC (4-dimethylaminocinnamaldehyde) method that has been described previously (Prior et al., 2010). A 1.0 g sample of Huiyuan leaves powder was added into 20 mL extraction solution, which was a mixture of acetone, deionized water, and acetic acid (150:49:1 v/v/v), placed on a shaker for 1.5 h and subsequently centrifuged at 4,500 rpm for 20 min at 20°C. The supernatant was collected for analysis at 620 nm in a quartz cell. Total proanthocyanidin concentrations of sample extracts were calculated as PACs = (C × D × V)/(1000 × S), where the total PACs are in mg/g; C is the concentration of PACs in a sample extract, in g/L; D is the dilution factor; V is the extraction volume, in milliliters; and S is the sample size, in grams.

### RNA Extraction, Library Construction, and Transcriptome Sequencing

The leaves collected after different light treatment were sent to Beijing Novogene Bioinformatics Technology Co., Ltd., for RNA-Seq. A total amount of 1 μg RNA per sample was used as input material for the RNA-seq library preparations. Sequencing libraries were generated using NEBNext^®^ Ultra™ RNA Library Prep Kit for Illumina^®^ (NEB, United States) following manufacturer’s recommendations and index codes were added to attribute sequences to each sample. The integrity and purity of the RNA samples were determined by 1% agarose gel electrophoresis, NanoPhotometer Spectrophotometer and Agilent 2100 Bioanalyzer. The clustering of the index-coded samples was performed on a cBot Cluster Generation System using TruSeq PE Cluster Kit v3-cBot-HS (Illumia) according to the manufacturer’s instructions. After cluster generation, the library preparations were sequenced on an Illumina Novaseq platform and 150 bp paired-end reads were generated. All raw sequence read data were deposited in the NCBI Short Read Archive (SRA) database under accession number SRR16019010 – SRR16019018.

### RNA-Seq Data Processing and Mapping of Reads to the Cotton Genome

Raw data (raw reads) of fastq format were firstly processed through perl scripts. Then clean data (clean reads) were obtained by removing reads containing adapter, reads containing ploy-N and low-quality reads. At the same time, Q20, Q30, and GC content of the clean data were calculated. All the downstream analyses were based on the clean data. Reference genome and gene model annotation files were downloaded from CottonGen^1^. The generated clean reads were mapped to the *G. hirsutum* (AD1) ‘TM-1’ genome ZJU-improved_v2.1_a1 using TopHat v2.0.12. The featureCounts v1.5.0-p3 was used to count the read numbers mapped to each gene (Liao et al., 2014). The clean read count is used to calculate the FPKM (expected number of fragments per kilobase of transcript sequence per millions base pairs sequenced), which is used to characterize gene transcription abundance (Bray et al., 2016).

### Analysis of Differentially Expressed Genes, Cluster Analysis, Gene Ontology and Kyoto Encyclopedia of Genes and Genomes Enrichment

Differential expression analysis was performed using the DESeq2 R package (version 1.16.1). Differentially expressed genes (DEGs) between the treated and control samples were identified with the criteria of false discovery rate (FDR) adjusted *p*-value < 0.05 and | log2 (Fold Change)| ≥ 1. The expression patterns of the DEGs between different samples were displayed using heat maps. Gene Ontology (GO) enrichment and KEGG pathway enrichment analysis of differentially expressed genes were implemented by the clusterProfiler R package, in which gene length bias was corrected. GO terms with corrected *p*-value less than 0.05 were considered as significantly enriched.

### Gene Set Enrichment Analysis

All expressed genes, regardless of whether or not they were differentially expressed, were used for GSEA analysis, which was performed using the Sangerbox tools with default parameters, a free online platform^2^. Gene set enrichment analysis (GSEA) sorts all genes in the comparison group according to the multiple of difference between groups, and then analyzes the up and down regulation of the whole set according to the sorted results. The enrichment score for each gene set is then calculated using the entire ranked list, which reflects how the genes for each set are distributed in the ranked list. Normalized enriched score (NES) was determined for each gene set, which defines the degree of enrichment. The significantly enriched gene set was selected based on nominal *p*-value of NES ≤ 0.05 and false discovery rate (FDR) *q*-value ≤ 0.25 (Subramanian et al., 2005).

### Validation of RNA-Seq Results With Quantitative Real-Time PCR

Real-time quantitative reverse transcription-PCR (qRT-PCR) was applied to verify the RNA-Seq results. Total RNA was isolated from leaves of Huiyuan treated by different lights and then used to generate cDNA. cDNA was synthesized by using an EASYspin Plus Plant RNA Kit (Aidlab Biotechnologies Co., Ltd.; Beijing China) with gDNA Eraser (Takara). Each reaction (final volume, 10 μL) contained 5 μL 2 × SYBR Green master mix (Roche), 0.5 μL of each the forward and reverse primer (10 μM), 1 μL of the cDNA template, and 3 μL of RNase free water. qRT-PCR was conducted in a LightCycler 480 system (Roche, United States) under the following parameters: 95°C for 5 min and 45 cycles of 95°C for 10 s, 58°C for 15 s, and 72°C for 20 s. The gene expression levels were calculated with the 2^–ΔΔ*T*^ method. The cotton *GhUBQ7* gene (*DQ116441*) was amplified as an internal control gene. For each tissue, three biological replicates each with three technical replicates were analyzed. The list of primers was shown in Supplementary Table 1.

### Sequence Alignment and Phylogenetic Analyses

The eight HY5 protein sequences from other plant species (Arabidopsis, tomato, rice, apple, grape, maize, pear, and peach) were obtained from the NCBI database^3^. Then, we used AtHY5 (AT5G11260) protein sequence as the query (*P* < 0.001) to search the cotton GhHY5 protein sequences using an online BLASTp in CottonGen (see Text Footnote 1) (Yu et al., 2014). Finally, a total of 22 members were considered as candidate genes of the *GhHY5* family. The protein sequences of the *GhHY5* genes were obtained from CottonGen (see Text Footnote 1). The sequence alignment was performed using DNAMAN software, and visualized using ESPript 3.0^4^. The phylogenetic analysis was conducted using MEGA 7.0 software, based on the ML (Maximum likelihood) algorithm implemented with a bootstrap value of 1000. Some raw data used in analysis of the expression profiles of *GhHY5s* in different tissues and treatments (YW, YB, HD, and HL) were obtained from unpublished transcriptome data. The gene expression heatmap was constructed using EvolView^5^.

### The Virus-Induced Gene Silencing System on Cotton Seedling

A 254 bp fragment of *GhHY5* (from 28 to 281 bp) was amplified using cDNA as template and cloned into the TRV2 vector. The forward and reverse primers contained *Eco*RI and *Kpn*I sites at the 5′ end, respectively. The primers are shown in Supplementary Table 1. TRV2-GhHY5, TRV2-GhCHLI (magnesium chelatase subunit I), TRV2, and pTRV1 were individually transformed into the *Agrobacterium tumefaciens* strain GV3101. The GV3101 culture of TRV2-GhHY5, TRV2-GhCHLI, TRV2, or TRV1 was incubated overnight at 28°C and resuspended to an OD600 of 0.8 in infiltration buffer containing 10 mM MgCl_2_, 10 mM MES, and 100 μM Acetosyringone (AS). Suspensions were kept at room temperature for 3 h without shaking. The TRV2-GhHY5 mixture (TRV1:TRV2-GhHY5 = 1:1, v/v) and the control mixture (TRV1:TRV2 = 1:1, TRV1:TRV2-GhCHLI = 1:1, v/v) were prepared for injection. The cotyledons of Huiyuan were selected to inject the VIGS constructs and 400 μl of suspension was injected vertically into each cotyledon. The infiltrated seedlings were kept in the dark overnight and then stored at 24°C with a 14/10 h day/night photoperiod in a growth chamber fitted with blue light. After 2 weeks, leaves were collected for anthocyanin measurement and RNA extraction.

### GUS Staining in *Nicotiana benthamiana* Leaves

The *GhPAP1-D* promoter fragment from Huiyuan (RP, 683 bp) or X61 (GP, 455 bp) was cloned and fused to the β-glucuronidase (GUS) reporter gene in *pBI121* vector using the *Hin*dIII and *Xba*I sites. The coding sequence of *GhHY5* was cloned into the *pCAMBIA2300* vector using the *Kpn*I and *Sal*I sites. *Agrobacterium* cultures (strain GV3101) containing the *35S:GhHY5* and the *pBI121* (*RP:GUS* or *GP:GUS*) vector with the promoter of *GhPAP1D* were transiently expressed in *N. benthamiana* leaves. *Agrobacterium* cultures carrying the constructs were suspended in infiltration buffer [10 mM MES, 10 mM MgCl_2_, 150 μM acetosyringone (AS), pH = 5.6] to an optimal density (OD600 = 0.5). After 3 days of infiltration, the expression of GUS was detected by histochemical staining as previously described (Jefferson et al., 1987). The GUS expression level from the infiltrated area was quantified using qRT-PCR as described above and this value was used to represent GUS activity level. The primers are shown in Supplementary Table 1.

### Luciferase Reporter Assay

The *GhPAP1-D* promoter fragment from Huiyuan (RP, 683 bp) or X61 (GP, 455 bp) was cloned and fused to the luciferase (*Luc*) reporter gene in *pGreenII 0800-LUC* vector (*RP:Luc* and *GP:Luc*) using the *Xho*I and *Pst*I sites. The coding sequence of *GhHY5* was cloned into the *pGreenII 62-SK* vector (*35S:HY5*) using the *Bsa*I and *Eco*31I sites. The *RP:Luc*, *GP:Luc* and *35S:HY5* constructs were transformed into *A. tumefaciens* strain GV3101. Transient expression of the vectors was carried out by agroinfiltration of *N. benthamiana* leaves and the subsequent dual luciferase assay was performed as described by Tao et al. (2018). The primers are shown in Supplementary Table 1.

## Results

### Light Quality Affects the Red Color Intensity of Huiyuan

**FIGURE 1:**
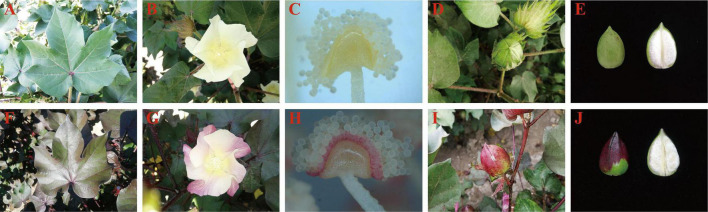
Phenotypic comparison between the green leaf cotton XinLuZao 61 (upper) and Huiyuan (bottom) grown in natural light. Panels **(A–E)** represent leaves, flowers, anthers, bolls, and fibers of X61, respectively. Panels **(F–J)** represent leaves, flowers, anthers, bolls, and fibers of Huiyuan, respectively.

The Huiyuan plant exhibits red pigment accumulation throughout its growing period in the natural environment. Compared to the control green leaf cultivar XinLuZao 61 (X61), the red phenotype of Huiyuan appears in all tissues and organs (such as steam, leaf, petal, anther wall, and cotton boll) throughout the life cycle of Huiyuan, except cotton fiber (Figure 1 and Supplementary Figure 1). In order to confirm that the red phenotype of Huiyuan is caused by anthocyanin accumulation, the total anthocyanin contents in hypocotyls, leaves and petals of green plant and Huiyuan were determined. As shown in Supplementary Figure 2, the total anthocyanin content in hypocotyls, leaves and petals of Huiyuan was significantly higher than that of green plants. These results indicate that the red phenotype of Huiyuan is a result of anthocyanin biosynthesis and accumulation. To determine whether Huiyuan plant is the same type as the previously reported *R1* or *RS* mutants, we cloned and sequenced the promoter and CDS regions of *GhPAP1A* (*Rs*, *GH_A07G0850*) and *GhPAP1D* (*R1*, *GH_D07G0852*) from Huiyuan. No difference was found in *GhPAP1A* between Huiyuan and *Rs*, but the same repeat sequence found in the promoter of *GhPAP1D* from *R1* was found in Huiyuan, although with a single base difference (Supplementary Figure 3).

**FIGURE 2:**
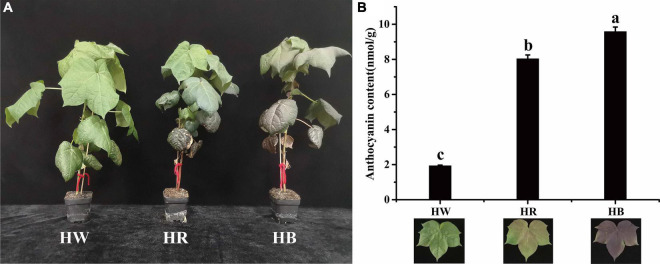
Cotton seedlings under different light treatments and their corresponding anthocyanin content. **(A)** Phenotypes of Huiyuan under different light treatments. **(B)** Anthocyanin content of Huiyuan leaves under different light treatments. HW, HR, and HB indicates samples treated by white light, red light, and blue light, respectively. Three biological replicates of each treatment were analyzed. Data are expressed as the means ± SD, *n* = 3. The letters on top of the bars indicate significance with the same letters being insignificant according to one-way analysis of variance (ANOVA) (*P* < 0.05).

An interesting phenomenon was found when Huiyuan plants were cultured in laboratory. Under laboratory white light (LED, light-emitting diode), Huiyuan plants showed green leaf phenotype similar to that of green cotton (Figure 2A). Considering that white LED light lacks a series of wavelengths compared with natural light, we added red light and blue light, respectively, on the basis of white light to investigate the effect on the expression of red phenotype. We also measured the total anthocyanin concentration in Huiyuan leaves after white light (HW), red light (HR), and blue light (HB) (Figure 2B). Compared to the control (HW), HR and HB significantly induced the expression of red color and anthocyanin accumulation with a greater effect observed for HB (Figure 2B).

### Huiyuan Transcriptome Sequencing and Analysis of Differentially Expressed Genes

To discern the molecular mechanism of anthocyanin biosynthesis and accumulation in Huiyuan under different lights, transcriptome sequencing was used to explore changes of gene expression in leaves of Huiyuan collected from plants treated with red light or blue light. We obtained approximately 54.04-65.89 million paired-end raw reads (Supplementary Table 2). Average clean data per sample was no less than 7.96 Gb (Supplementary Table 2). 92.16-94.07% of the total clean reads were uniquely mapped to the TM-1 reference genome (see section “Materials and Methods” for details) with 72.87–77.26% mapped to exons (Supplementary Table 2). Pearson correlation coefficient [the square of Pearson correlation coefficient (*R*^2^) in the sample group was greater than 0.95 and greater than that between groups] and principal component analysis (PCA) calculated based on the FPKM values of all genes in each sample showed significant differences between groups and good intra-group reproducibility (Supplementary Figure 4). To further confirm the accuracy of transcriptomic data, we selected 16 differentially expressed genes of the anthocyanin biosynthesis pathway for qRT-PCR verification. Although there were slight differences between the results of RNA-seq and qRT-PCR, the overall trend was consistent, indicating the reliability of transcriptomic data (Supplementary Figure 5).

**FIGURE 3:**
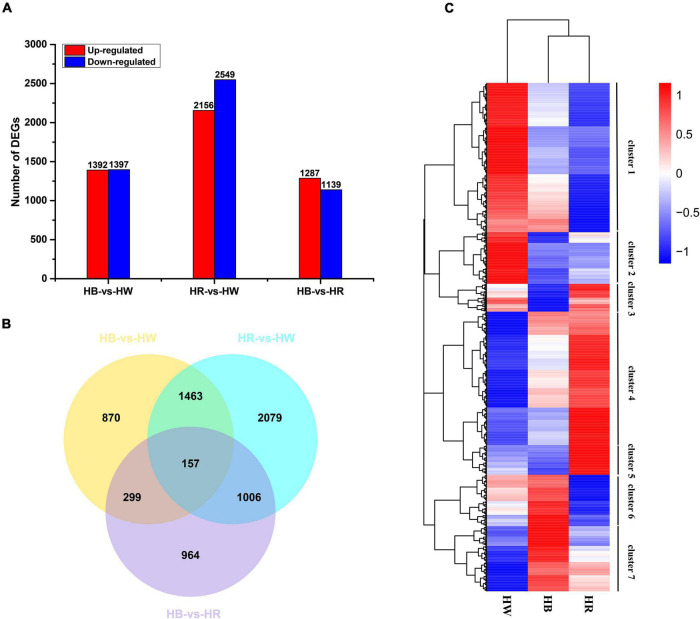
Differential gene expression induced by red or blue light. **(A)** Numbers of DEGs in pairwise comparisons of the three libraries. **(B)** Venn diagram showing DEG distributions. **(C)** Expression profile clustering. The color scale at the right represents re-processed log10 (FPKM + 1) using heatmap, representing the relative expression level. The expression variance for each gene is indicated by different colors ranging from low (blue) to high (red). HW, HR, and HB indicates samples treated by white light, red light, and blue light, respectively.

Based on a false discovery rate (*p*adj < 0.05) and |log2(FoldChange)| > 1, pairwise comparisons identified a total of 9920 DEGs amongst different light treatments, with 2789 (1,392 up-regulated and 1,397 down-regulated), 4,705 (2,156 up-regulated and 2,549 down-regulated), and 2,426 (1,287 up-regulated and 1,139 down-regulated) in the comparison of HB-vs.-HW, HR-vs.-HW, and HB-vs.-HR, respectively. Compared with other comparison groups, the HR-vs.-HW had the largest number of differentially expressed genes (Figure 3A). Of the DEGs, 157 were common in all three comparisons, and 870, 2,079, and 964 genes were specific in HB-vs.-HW, HR-vs.-HW, and HB-vs.-HR, respectively (Figure 3B). For a global view of the expression patterns of DEGs, the 6,838 DEGs (non-redundant DEGs amongst the three comparisons) in the three pairwise comparisons were further analyzed by hierarchical cluster analysis (Figure 3C). The genes could be clustered into 7 groups with their expression pattern being (1) HW > HB > HR, (2) HW > HR > HB, (3) HR > HW > HB, (4) HR > HB > HW, (5) HR > HW > HB, (6) HB > HW > HR, and (7) HB > HR > HW. In addition, the result showed clustering of HR and HB, indicating their similarities in gene expression.

### Analysis of Functional Enrichment of Differentially Expressed Genes

To understand the function of differentially expressed genes, Gene Ontology (GO) enrichment analysis was carried out with the DEGs in the pairwise comparison of three groups (HB-vs.-HW, HR-vs.-HW, and HB-vs.-HR). The GO annotation for DEGs of HB-vs.-HW and HR-vs.-HW revealed numerous common GO subcategories significantly enriched in three categories, i.e., Biological Process (BP), Molecular Function (MF), and Cellular Component (CC) (Supplementary Figure 6 and Supplementary Tables 3–5), such as ‘photosystem,’ ‘membrane protein complex,’ ‘thylakoid’ in CC category; ‘oxidoreductase activity,’ ‘drug transporter activity,’ ‘chlorophyllide a oxygenase activity’ in MF category; ‘photosynthesis,’ ‘cellular carbohydrate metabolic process,’ ‘disaccharide metabolic process’ in BP category. Some *MATE* genes in the ‘drug transporter activity’ pathway may be closely related to anthocyanin transport. In addition, the DEGs in HB-vs.-HR were mainly enriched with the GOs of ‘movement of cell or subcellular component,’ ‘DNA replication,’ ‘circadian rhythm’ (the genes in the category of ‘circadian rhythm’ are involved in the perception and transduction process of organisms to light signals), and ‘sucrose metabolic process’ (sucrose is often considered as a signal molecule for anthocyanin biosynthesis) of the BP category (Supplementary Figure 6 and Supplementary Table 5). It is worth noting that the expression of *HY5* (a positive light response regulator) in the ‘circadian rhythm – plant’ pathway showed an up-regulation under red (2.3-fold) and blue (1.9-fold) light treatment. In addition, we found that many sucrose synthesis and modification genes were enriched with ‘sucrose metabolic process.’

**FIGURE 4:**
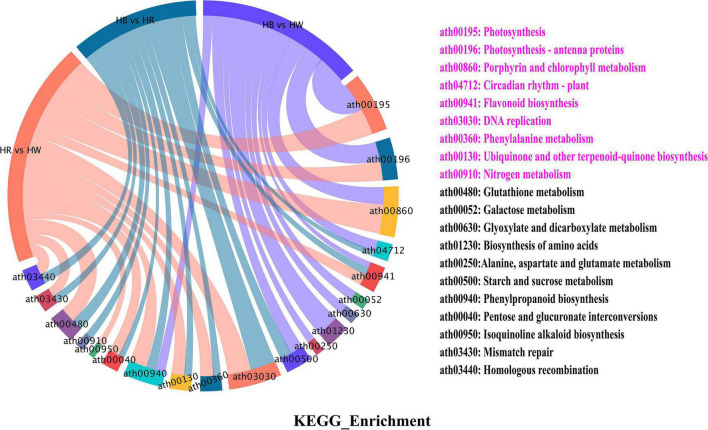
KEGG enrichment of all differential genes (DEGs). The left side of the figure is the schematic diagram of the enriched categories of different genes in different comparison groups. On the right side of the figure are the notes of the enriched categories, and the pink text indicates the significantly enriched categories. HW, HR, and HB indicates samples treated by white light, red light, and blue light, respectively.

We executed KEGG (Kyoto Encyclopedia of Genes and Genomes) pathway enrichment analysis to gain further insight into related pigmentation metabolic pathway of DEGs. The 6838 DEGs were enriched with 93 biosynthesis and metabolism pathways, among those were ‘phenylalanine metabolism,’ ‘flavonoid biosynthesis,’ ‘phenylpropanoid biosynthesis,’ and ‘glutathione metabolism’ involved in the flavonoid pathway (Figure 4 and Supplementary Table 6). As expected, we found two *PAL* (*GH_A01G2316*, *GH_D11G3728*), one C4H (*GH_D10G2039*), and one *4CL* (*GH_D03G0525*) genes to be up-regulated with red and blue light radiation, and to be enriched in ‘phenylalanine metabolism’ and ‘phenylpropanoid biosynthesis’ pathway (Supplementary Table 14). Some structural genes (*CHS*, *CHI*, *F3H*, *F3′H*, *F3′5′H*, and *FLS*) involved in the biosynthesis of flavonoids, flavonols, and anthocyanins were also significantly up-regulated, and enriched in the ‘flavonoid biosynthesis’ pathway under red and blue light treatment (Supplementary Tables 7, 8). Furthermore, we found that ‘photosynthesis,’ ‘DNA replication,’ ‘circadian rhythm – plant,’ and ‘nitrogen metabolism’ were significantly enriched in different the comparison group (Supplementary Tables 7–9 and Supplementary Figure 7).

### Gene Set Enrichment Analysis

**FIGURE 5:**
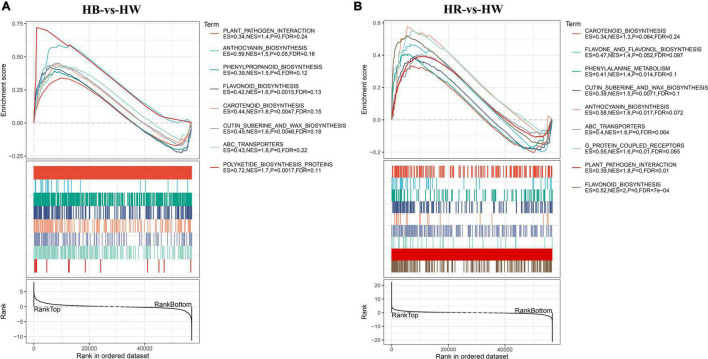
Gene set enrichment analysis (GSEA). GSEA was performed in the HB-vs-HW **(A)** and HB-vs-HW **(B)** groups. Gene sets with a normalized enrichment score (NES) > 1 and FDR < 0.25 were considered as statistically significant. HW, HR, and HB indicates samples treated by white light, red light, and blue light, respectively.

A minority of key genes in GO and KEGG enrichment analysis may be neglected by an arbitrary cutoff on the basis of fold-change or significance. Thus, we implemented gene set enrichment analysis (GSEA) to further investigate the functional alterations correlated with blue or red radiation treatment (HB-vs.-HW and HR-vs.-HW). GSEA was performed using a GO-based list (9,044 gene sets) and a KEGG-based list (196 gene sets). The lists of all up regulated gene sets (GO and KEGG gene sets) are provided in Supplementary Tables 10–13. GO enrichment analysis by GSEA showed that the gene sets with a higher expression level were involved in ‘Flavone metabolic process,’ ‘Flavonol metabolic process,’ ‘ABC type transporter activity,’ ‘Cinnamic acid metabolic process,’ ‘Phenylpropanoid biosynthetic process,’ and ‘Proanthocyanidin biosynthetic process’ (Supplementary Tables 10, 11), which was very different from the results based on the GO enrichment analysis (Supplementary Tables 3, 4). Some *ABC* genes that may be involved in anthocyanin accumulation were significantly enriched in the ‘ABC type transporter activity’ under red and blue light treatment. It is worth pondering that the *LAR* (*GH_A12G1894*, *GH_A12G2855*, *GH_D12G1894*, *GH_D12G2880*) and *MATE* (*GH_A09G0080*, *GH_D09G0085*, *GH_A12G0833*, *GH_D12G0748*) genes in the ‘Proanthocyanidin biosynthetic process’ were significantly differentially expressed only under red light. Correspondingly, the red light contributed more to proanthocyanidins accumulation (Supplementary Figure 8). In addition, from the KEGG-based list, the higher expression gene sets in HB or HR group were mainly related to ‘Polyketide biosynthesis proteins,’ ‘Cutin suberine and wax biosynthesis,’ ‘Carotenoid biosynthesis,’ ‘Anthocyanin biosynthesis,’ and ‘Plant pathogen interaction,’ which were not significantly enriched by the classical KEGG analysis (Figure 5 and Supplementary Tables 12, 13). Interestingly, in addition to the two MYB genes (*GhPAP1A* or *GH_A07G0850* and *GhPAP1D* or *GH_D07G0852*) and *GhGSTF12* (*GH_A07G0814/GH_A07G0816*) previously reported (Li et al., 2019; Liang et al., 2020; Shao et al., 2021), other *MYB*, *3GT*, and *GST* genes might be involved in anthocyanin biosynthesis as they were included in the ‘Anthocyanin biosynthesis’ pathway. On the other hand, ‘Circadian rhythm,’ ‘DNA replication,’ ‘Photosynthesis,’ and ‘Nitrogen metabolism’ showed no enrichment according to GSEA analysis (Supplementary Tables 7, 8, 12, 13).

In addition to the above significantly enriched genes, many differentially expressed genes that may be involved in anthocyanin biosynthesis were identified based on gene function annotation (Supplementary Table 14). Firstly, several photoreceptor factors, such as cryptochrome (*CRY2*, *GH_D02G0431*), phytochrome (*PHYA*, *GH_A11G2938*), phototropins (*PHOT1*, *GH_A05G1007* and *GH_D03G1511*; *PHOT2*, *GH_A12G0110*), and UVR8 (*GH_A10G2465* and *GH_D10G2574*) were differentially expressed under red and blue light. Secondly, numerous MYB, bHLH, and WD40 TFs were amongst the DEGs, implying that their regulatory role could be influenced by the blue or red light.

Plant hormones have been found to regulate anthocyanin biosynthesis (Das et al., 2012). We found several auxin-responsive elements, such as *ARFs* and *SAURs*, were enriched in categories ‘response to hormone’ (GO:0009725) and ‘response to auxin’ (GO:0009733) in both red and blue light treatments, which signifies that auxin could play a role in light induced anthocyanin biosynthesis in Upland cotton leaves. In addition, some genes encoding *DELLA* and *JAZ* proteins (the key factors of the GA and JA signal transduction pathway, respectively), which directly interact with bHLHs and MYBs in the MBW complex (Qi et al., 2011; Xie et al., 2016), were DEGs. In summary, these results support the role of red or blue light in regulation of genes related to anthocyanin biosynthesis and accumulation.

### Analysis of Differentially Expressed Genes Associated With *GhHY5s* in Different Tissues of Upland Cotton

**FIGURE 6:**
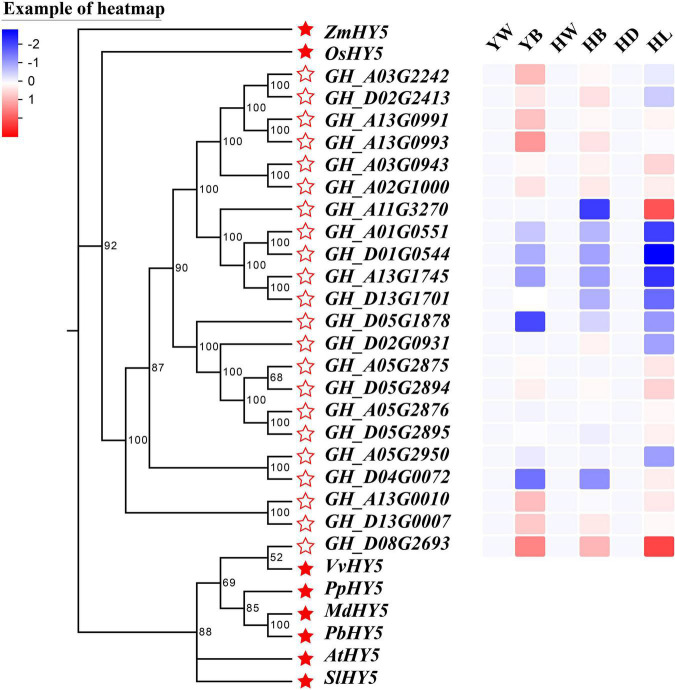
Phylogenetic tree of *GhHY5s* and the reported *HY5s* in other plants, and the expression profile of *GhHY5s* from different tissues under different lighting conditions. The GenBank accession numbers for the HY5 from other plants are AtHY5 (BAA21327.1), SlHY5 (CAB57979.1), OsHY5 (BAD35451.1), MdHY5 (BAM71071.1), VvHY5 (AGX85877.1), ZmHY5 (AQK69393.1), PbHY5 (QGP73826.1), and PpHY5 (ONI34365.1). Scale bar indicates the expression level, from low expression (blue) to high expression (red). YW, green leaves of *Rs* under white light (LED); YB, red leaves of *Rs* under blue light (white LED light plus blue light); HW, green leaves of *R1* under white light (LED); HB, red leaves of *R1* under blue light (white LED light plus blue light); HD, white hypocotyls of Huiyuan under dark; HL, red hypocotyls of Huiyuan under light (LED).

In recent decades, a large number of studies have found that some essential light-responsive transcription factors, such as HY5, BBX, COP1, and PIF, can affect anthocyanin biosynthesis under UVs, red or blue light treatment (Zhang et al., 2011; Shin et al., 2013; Liu et al., 2015, 2018; Jiang et al., 2016; An et al., 2017; Tao et al., 2018; Bai et al., 2019c; Kim et al., 2020). We found a differentially expressed *HY5* to be one of the genes of the enriched GO term ‘circadian rhythm.’ In order to more comprehensively identify HY5 that may be involved in the regulation of anthocyanin biosynthesis, we did a genome-wide identification of *HY5* genes in *G. hirsutum* and found 22 candidate *GhHY5* genes. Based on phylogenetic analysis of GhHY5 together with HY5 proteins from other plants (Figure 6), *GH_D08G2693*, the gene found in the enriched GO term ‘circadian rhythm’, was the most closely related to HY5 from other di-cotyledon plants, such as Arabidopsis, grapes and tomatoes, and distinct from the HY5 of maize and rice. We then compared the transcriptional level of *GH_D08G2693* (hereafter *GhHY5*) in hypocotyls and leaves of red leaf cotton cultivars (*R1/*Huiyuan and *Rs*). As expected, the expression level of *GhHY5* was highly correlated with the appearance of the red phenotype (Figure 6), implying a role of *GhHY5* in the coloration of cotton plants.

### Silencing of *GhHY5* by Virus-Induced Gene Silencing

**FIGURE 7:**
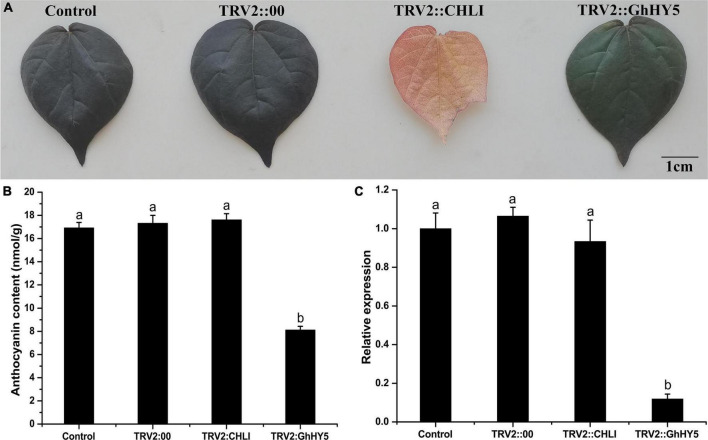
Comparison of leaf color, relative expression level of *GhHY5* and anthocyanin contents in the leaves from the VIGS assay. **(A)** Effect of TRV-VIGS silencing of *GhHY5* and *GhCHLI* in Huiyuan plants on leaf color phenotypes. Control was the untreated Huiyuan leave. TRV2:00 is empty vector. Other two leaves were taken from infiltrated plants 2 weeks post-treatment with the corresponding construct. Scale bar: 1 cm. **(B)** Anthocyanin concentration in leaves from plants under different treatments. **(C)** qRT-PCR analysis of *GhHY5* expression in leaves from plants under different treatments. *GhUBQ7* was used as a control gene. Three biological replicates of each treatment were analyzed. Data are expressed as the means ± SD, *n* = 3. The letters on top of the bars indicate significance with the same letters being insignificant according to one-way analysis of variance (ANOVA) (*P* < 0.05).

As expression analysis indicated that the elevated expression of *GhHY5* could be responsible for anthocyanin biosynthesis (Figure 6), we speculated that silencing of *GhHY5* in Huiyuan would reduce its anthocyanin level. So, we used virus-induced gene silencing (VIGS) to test this hypothesis. Silencing of *GhHY5* in Huiyuan decreased its expression level (Figure 7C) and the content of anthocyanins and the red color intensity compared with controls (Figures 7A,B). The transcription of other genes of the anthocyanin biosynthesis pathway was affected to varying degrees due to the down-regulation of *GhHY5* (Supplementary Figure 9). These results suggest a role of *GhHY5* in accumulation of the anthocyanin in cotton leaves.

### Regulatory of *GhHY5* on *GhPAP1D*

**FIGURE 8:**
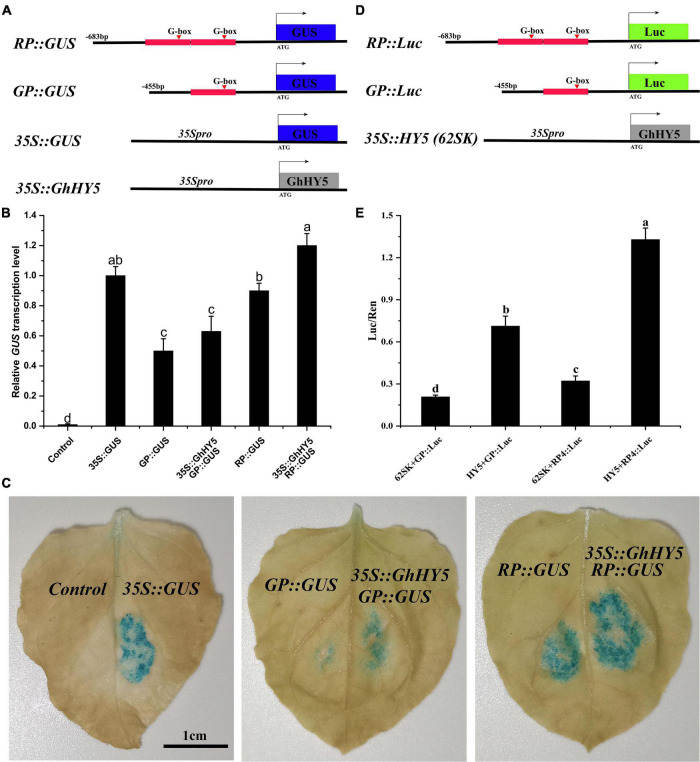
GhHY5 act as a *cis*-regulator of *GhPAP1D* expression. **(A)** Schematic diagram of constructs used in GUS experiment. **(B)** qRT-PCR analysis was conduct to detect GUS expression using three biological replicates. **(C)** GUS staining of *35S:GUS*, *GP:GUS*, *RP:GUS*, *GP:GUS* plus *35S:GhHY5*, and *RP:GUS* plus *35S:GhHY5*. **(D)** Schematic diagram of constructs used in the luciferase reporter assay. **(E)** Effects of GhHY5 on the activity of RP and GP promoters of *GhPAP1D* in luciferase assays. The ratio of Luc/Ren of the empty vector (62SK) plus promoter was used as the control. Different letters denote significant differences according to one-way analysis of variance (ANOVA) (*P* < 0.05).

To determine whether *GhHY5* regulates the expression of *GhPAP1D*, we generated GUS expression constructs driven by the *GhPAP1D* promoter from Huiyuan (683 bp, RP) and X61 (455 bp, GP). The 228 bp repeat in the *GhPAP1D* promoter of Huiyuan was thought be important for the function of *GhPAP1D* in anthocyanin biosynthesis and accumulation due to the presence (at position −369 bp) of an additional G-box, the potential binding site of GhHY5. The GUS construct with the RP or GP fragment was introduced into *N. benthamiana* leaves and the GUS activity was measured (Figure 8). The results showed that the level of GUS activity was greater for the *RP:GUS* than for *GP:GUS* (Figure 8B). Compared to *RP:GUS* or *GP:GUS* alone, co-expressing *35S:GhHY5* with the *RP:GUS* or *GP:GUS* increased GUS activity and relative level of GUS, particularly when co-expressing with *RP:GUS* (Figures 8B,C). For the LUC assay, we constructed the *RP:Luc* and *GP:Luc* reporter and the effectors *35S:HY5* (Figure 8D). Consistent with the GUS experimental results, RP has a stronger driving effect on expression of *Luc* gene than GP in the absence of HY5, which may be attributed to the repeat region on the RP (Figure 8E). In addition, the expression of the *Luc* gene driven by the RP and GP promoter was significantly enhanced when it was co-expressed with GhHY5 compared with control (62SK + *GP:Luc* or 62SK + *RP:Luc*), and the change of HY5 + *RP:Luc* is greater than that of HY5 + *GP:Luc* (Figure 8E). In short, these results suggested that GhHY5 is able to activate *GhPAP1D* expression through the G-box site.

## Discussion

Studies in a variety of specie have shown that specific light spectral wavelengths can affect the biosynthesis of anthocyanins. Generally, UV – B and blue light are considered to be the main light sources affecting anthocyanin accumulation (Xu et al., 2014; Tao et al., 2018; Kim et al., 2020; Li et al., 2021). Besides, evidence suggests that red light also seems to play a positive role in anthocyanin biosynthesis (Zhang et al., 2018; Samkumar et al., 2021). Interestingly, red light can promote anthocyanin accumulation more than blue light in bilberry, but it is the opposite in strawberry (Lekkham et al., 2016; Zhang et al., 2018; Samkumar et al., 2021). In this study, we used a comparative transcriptomics approach to analyze the effect of red and blue light on anthocyanin biosynthesis and the genes involved in regulation of the effect using leaves of Huiyuan, a red Upland cotton accession with the same mutation as that found in the red leaf mutant *R1*. The highest leaf anthocyanin concentration in Huiyuan was observed under blue light followed by red light, consistent with the results reported in grape, strawberry, and bilberry fruit (Kondo et al., 2014; Zhang et al., 2018; Samkumar et al., 2021). We also identified DEGs under different light treatments and demonstrated a potential role of *GhHY5* in regulation of anthocyanin synthesis and accumulation.

### Plant Light Signal Perception and Transduction

In the natural environment, plants perceive and transmit light signals through photoreceptors, such as PHYs, CRYs, PHOTs, and UVR8 (Jiang et al., 2016; Zhang et al., 2016; Tao et al., 2018; Ma et al., 2019). In this study, we found that cryptochrome, phytochrome, and phototropins, except for two *UVR8*, were down-regulated under blue and red light conditions (Supplementary Table 14). The down-regulation of several photoreceptor related genes is consistent with previous studies. which may be considered a hallmark of organization coloring completion (Kadomura-Ishikawa et al., 2013; Zhang et al., 2018). Besides, the regulation of anthocyanin synthesis via CRYs requires interplay with the activity of photoreceptor for regulating the expression of downstream genes under the natural conditions (Ahmad and Cashmore, 1997; Ahmad et al., 1998; Mas et al., 2000; Morales et al., 2013; Jenkins, 2014). This may be the reason for the up-regulation of *UVB8* induced by blue light. Noticeably, several photoreceptor factors and regulatory factors (such as *CRY2*, *PHYA*, *PHOTs*, *DELLA*, and *JAZ*) had a low transcription abundance in leaves treated with red and blue light. However, the high anthocyanin content was detected in red and blue light treatment. Possibly, these genes decreased at this stage that anthocyanin concentration reached saturation, suggesting that light signal perception and transduction components were dynamic to keep the regulatory factors and structural genes of anthocyanin biosynthesis at a high expression level in red and blue light treatment. Interestingly, *GhHY5* (*GH_D08G2693*) was one of genes significantly enriched in ‘circadian rhythm’ category of GO and KEGG analyses (Figure 4, Supplementary Figure 7, and Supplementary Tables 3, 4, 6). Numerous previous studies have proved that HY5 is involved in the regulation of anthocyanin biosynthesis in various species (Nguyen et al., 2015; An et al., 2017; Liu et al., 2018; Tao et al., 2018; Wang et al., 2020; Zhao et al., 2020b). We showed that *GhHY5* is also involved in the regulation of anthocyanin biosynthesis in cotton leaves. Firstly, the phylogenetic analysis indicated that *GhHY5* is closely related to *HY5* that has a demonstrated role regulation of anthocyanin biosynthesis in other plant species (Figure 6). Secondly, virus-induced silencing of *GhHY5* in cotton seedlings significantly hold back the formation of red coloration and decreased the content of anthocyanins (Figure 7). However, the silencing of *GhHY5* expression did not cause sharp decline of anthocyanin concentration (Figures 7A,B), suggesting the existence of HY5-independent anthocyanin biosynthesis pathway (Qiu et al., 2019). To clarify the regulatory mechanism of *GhHY5* in anthocyanin biosynthesis, we performed luciferase reporter assay and GUS assay to determine the effect of the GhHY5 protein on the expression of *GhPAP1D*, which has been reported to be involved in the regulation of anthocyanin biosynthesis in Upland cotton (Gao et al., 2013; Li et al., 2019). These results suggested that the regulation of GhHY5 on the expression of *GhPAP1D* may be through the G-box located in the promoter of *GhPAP1D* (Figures 8B,C,E). Furthermore, the number of G-box could influence the regulation of *GhPAP1D* expression by GhHY5 as a higher GUS and luciferase activity was observed in *RP:GUS/RP:Luc* (with two G-boxes) than in *GP:GUS/GP:Luc* (with a single G-box) (Figures 8B,E).

### Genes Related to Anthocyanin Biosynthesis and Regulation

The anthocyanin biosynthesis is regulated by the well-known MBW regulatory complex composed of MYB, basic-helix-loop-helix (bHLH), and WD40 repeat families (Zhang B. et al., 2019). Previous studies have shown that *GhPAP1A* (*GH_A07G0850*) and *GhPAP1D* (*GH_D07G0852*) are the key regulators activating anthocyanin biosynthesis in Upland cotton (Li et al., 2019; Liang et al., 2020). In this study, *GhPAP1D* was up-regulated by red and blue light. In addition, two cluster of MYB genes were found on chromosomes A07 (*GH_A07G0849*, *GH_A07G0850*, and *GH_A07G0851*) and D07 (*GH_D07G0852* and *GH_A07G0853*). These genes were found in the ‘Anthocyanin biosynthesis’ pathway by GSEA analysis.

As a branch of flavonoid biosynthesis pathway, most structural genes of anthocyanin biosynthesis have been deeply studied in numerous plant species (Chaves-Silva et al., 2018). In the present study, RNA-seq analysis has shown that many candidate anthocyanin structural genes (*PAL*, *C4H*, *4CL*, *CHS*, *CHI*, *F3H*, *F3′H*, *F3′5′H*, *DFR*, *ANS*, and *3GT*) were up-regulated under red and blue light treatment. Besides, consistent with previous studies, some proanthocyanidin (PA) biosynthesis related genes (*LAR*; *GH_A12G1894*, *GH_A12G2855*, *GH_D12G1894*, and *GH_D12G2880*) were significantly up-regulated under red light treatment compared with blue light treatment (Supplementary Table 14) (Zhang et al., 2018). Activation of proanthocyanidins synthesis pathway under red light could shunt the substrates required for anthocyanin synthesis by inducing the *LARs* and *MATEs*, which could explain the difference of the red color intensity under red and blue light conditions (Supplementary Figure 8). Sucrose as a signal molecule involved in anthocyanin biosynthesis has been reported in many species (Solfanelli et al., 2006; Das et al., 2012). In our study, we found that many sucrose synthesis and modification genes were DEGs in the HB-vs-HW comparison. The significant enrichment of ‘sucrose synthase activity’ (GO:0016157) category only under blue light might also be one of the reasons contributing to the high anthocyanin concentration after blue light treatment (Supplementary Figure 6 and Supplementary Table 3).

### Transporters Involved in Anthocyanin Transport

Whether the anthocyanins synthesized in the cytoplasm can be smoothly transported into the vacuole plays an important role in the coloring of plant tissues and organs. So far, the molecular mechanism of anthocyanin transport has been partially revealed, mainly involving transporters such as *MATEs*, *ABCs* and *GSTs* (Zhao and Dixon, 2010). Among them, the involvement of *GST* in anthocyanin transport and accumulation has been proved in multiple species such as corn (Pairoba and Walbot, 2003), petunia (Alfenito et al., 1998), Arabidopsis (Kitamura et al., 2004), peach (Zhao et al., 2020a), apple (Jiang et al., 2019), and kiwifruit (Liu et al., 2019). Interestingly, in addition to *GhGSTF12* (Shao et al., 2021), many other *GST* genes were differentially expressed under red and blue light treatment (Supplementary Table 14). In addition, a large number of *MATE* and *ABC* genes were differentially expressed and enriched in ‘drug transmembrane transport’ (Supplementary Figure 6 and Supplementary Tables 3, 4) and ‘ABC-transporters’ pathway (Figure 5 and Supplementary Tables 12, 13) under red and blue light treatment, respectively. It is noteworthy that several *MATE* genes (*GH_A09G0080*, *GH_D09G0085*, *GH_A12G0833*, and *GH_D12G0748*) involved in proanthocyanidin transport (Xu et al., 2019) were up-regulated and enriched in ‘Proanthocyanidin biosynthesis process’ (Supplementary Table 13) under red light treatment but not under blue light treatment (Supplementary Table 14). This result also shows that red light activated biosynthesis pathway of proanthocyanidins.

## Conclusion

In this study, RNA-seq analysis of a red Upland cotton accession showed that both red and blue light could induce numerous DEGs involved in phenylpropane (*PAL*, *C4H*, and *4CL*), flavonoids (*CHI*, *CHs*, *F3H*, *F3′H*, *F3′5′H*, and *FLS*), proanthocyanidins (*LAR* and *MATE*), anthocyanins (*ANS*, *UFGT*, and GST) pathway. Red and blue light treatment can actively up regulate major genes related to anthocyanin biosynthesis, but blue light had a stronger effect on anthocyanin accumulation than red light. In addition, based on transcriptome analysis, VIGS, Luciferase and GUS assays, we found that *GhHY5* is involved in the accumulation of light-induced anthocyanins in Upland cotton leaves potentially by regulating the expression of *GhPAP1D*. Our results shed insight on the role of different lights in regulation of anthocyanin biosynthesis and accumulation in cotton.

## Data Availability Statement

The original contributions presented in the study are publicly available. This data can be found here: National Center for Biotechnology Information (NCBI) BioProject database under accession number PRJNA765172.

## Author Contributions

The presented study was conducted in collaboration by all authors. DS, FX, and JS conceived and designed the experiments. DS, FX, QL, XW, and YL performed the experiments. DS, XZ, YS, FL, and FX analyzed the data. DS, Q-HZ, FX, and JS wrote the manuscript.

## Conflict of Interest

The authors declare that the research was conducted in the absence of any commercial or financial relationships that could be construed as a potential conflict of interest.

## Publisher’s Note

All claims expressed in this article are solely those of the authors and do not necessarily represent those of their affiliated organizations, or those of the publisher, the editors and the reviewers. Any product that may be evaluated in this article, or claim that may be made by its manufacturer, is not guaranteed or endorsed by the publisher.

## Footnotes

1
http://www.cottongen.org

2
http://www.sangerbox.com/tool

3
https://www.ncbi.nlm.nih.gov/

4
https://espript.ibcp.fr/ESPript/cgi-bin/ESPript.cgi

5
https://evolgenius.info//evol/evolStat.php
