# Supplementary material for: Transcriptome Analysis Reveals Differences in Anthocyanin Accumulation in Cotton (Gossypium hirsutum L.) Induced by Red and Blue Light
Source: Front Plant Sci. 2022 Mar 31;13:788828. doi: 10.3389/fpls.2022.788828 (PMC9009209; doi:10.3389/fpls.2022.788828)
Supplement: Supplementary file 1 [file Table_1.DOCX]

**Table S1.** The primers sequences used in this article

| V_HY5_F | GAATTCGCAGCTAGTTCCTTACCTTC | for VIGS experiments |
| --- | --- | --- |
| V_HY5_R | GGTACCCTCTTTAAGCGCTTGTTTTC |  |
| 2413P4_F | AAGCTTACTTTAACCTCCCAAAAT | for GUS experiments |
| 2413P_R | TCTAGAATAAAAGCTGTTATAGTTAGCTTGC |  |
| HY5_F | GGTACCATGCAAGAACAAGGAACGAG | for GUS experiments |
| HY5_R | GTCGACTTAAAGGGTTCCATCAGCTG |  |
| P4_F (*Xho* Ⅰ) | CTCGAGACTTTAACCTCCCAAAAT | for Luciferase assay |
| P_R (*Pst Ⅰ*) | CTGCAGATAAAAGCTGTTATAGTTAGCTTGC |  |
| HY5_F_1 | ATGCAAGAACAAGGAACGAG | for Luciferase assay |
| HY5_R_1 | TTAAAGGGTTCCATCAGCTG |  |
| Q_HY5_F | TTCACTGACCCGACCGGA | for qRT-PCR experiments |
| Q_HY5_R | GATACTCTGTTCCTCAACAACCTCT |  |
| Q_CHS_F | CAGGAGAAGGACTGGAGTGG | for qRT-PCR experiments |
| Q_CHS_R | AGCAGCAACACTATGGAGCA |  |
| Q_CHI_F | ATGGAGTTTCTCCTCCAGCA | for qRT-PCR experiments |
| Q_CHI_R | GGTTTTTCACTGTCGACTCCA |  |
| Q_F3H_F | CTGAAGAAGCTGGCCAAAGA | for qRT-PCR experiments |
| Q_F3H_R | TGCAAGGATTTCCTCCAATG |  |
| Q_F3'H_F | GCTGATGTTAGGGGCAATGA | for qRT-PCR experiments |
| Q_F3'H_R | CTCACCATGAAACGACAACG |  |
| Q_F3'5'H_F | AAACATGGATGAGGCCTTTG | for qRT-PCR experiments |
| Q_F3'5'H_R | GCAAGGGATGTGCTTAGGAA |  |
| Q_DFR_F | CATGTTCGTAGGAGCTGTCG | for qRT-PCR experiments |
| Q_DFR_R | GGTAGGCACTCAATTGTTGAAA |  |
| Q_ANS_F | GCCACCGAAGGATAAGATCA | for qRT-PCR experiments |
| Q_ANS_R | TGGGTCTTCCTGAACAGCTT |  |
| Q_ANR_F | TGGGATCGAGGAAATCTACG | for qRT-PCR experiments |
| Q_ANR_R | ACCATAATCATTGGGGAAGC |  |
| Q_LAR_F | GAATGAGCCATTCCGAACAT | for qRT-PCR experiments |
| Q_LAR_R | GCTTCGACTACTGGCTTTGG |  |
| Q_3GT_F | AAGAAAGCGGAGCTAGTGTTCATCC | for qRT-PCR experiments |
| Q_3GT_R | GGAGATGGAGAGGTTGGAATTGAGA |  |
| Q_UBQ7_F | GAAGGCATTCCACCTGACCAAC | for qRT-PCR experiments |
| Q_UBQ7_R | CTTGACCTTCTTCTTGTGCTTG |  |
| Q_PAP1D_F | ACGTGGCATTAGCTAACGAC | for qRT-PCR experiments |
| Q_PAP1D_R | TTCCCACCACATCATCTCATCG |  |
| Q_PAL_F | AGCTTGGAACTGGGTTGTTG | for qRT-PCR experiments |
| Q_PAL_R | AGCACCATTCCAACCCTTTA |  |
| Q_C4H_F | TAGTCTCTAAACTTCGTGGCAAGCG | for qRT-PCR experiments |
| Q_C4H_R | GGTGGTTCAAGTCATCGCCGA |  |
| Q_4CL_F | AAGGTGCACTTTGTTCATGC | for qRT-PCR experiments |
| Q_4CL_R | CGTTGCAATTTAAAAGCCAAAT |  |
| Q_GSTF12D_F | ACATCTACGAGCAACGCTTG | for qRT-PCR experiments |
| Q_GSTF12D_R | ATGTGCCACATCCCAACATC |  |
