# Supplementary material for: Transcriptome Analysis Reveals Differences in Anthocyanin Accumulation in Cotton (Gossypium hirsutum L.) Induced by Red and Blue Light
Source: Front Plant Sci. 2022 Mar 31;13:788828. doi: 10.3389/fpls.2022.788828 (PMC9009209; doi:10.3389/fpls.2022.788828)
Supplement: Supplementary file 15 [file Data_Sheet_1.docx]

**
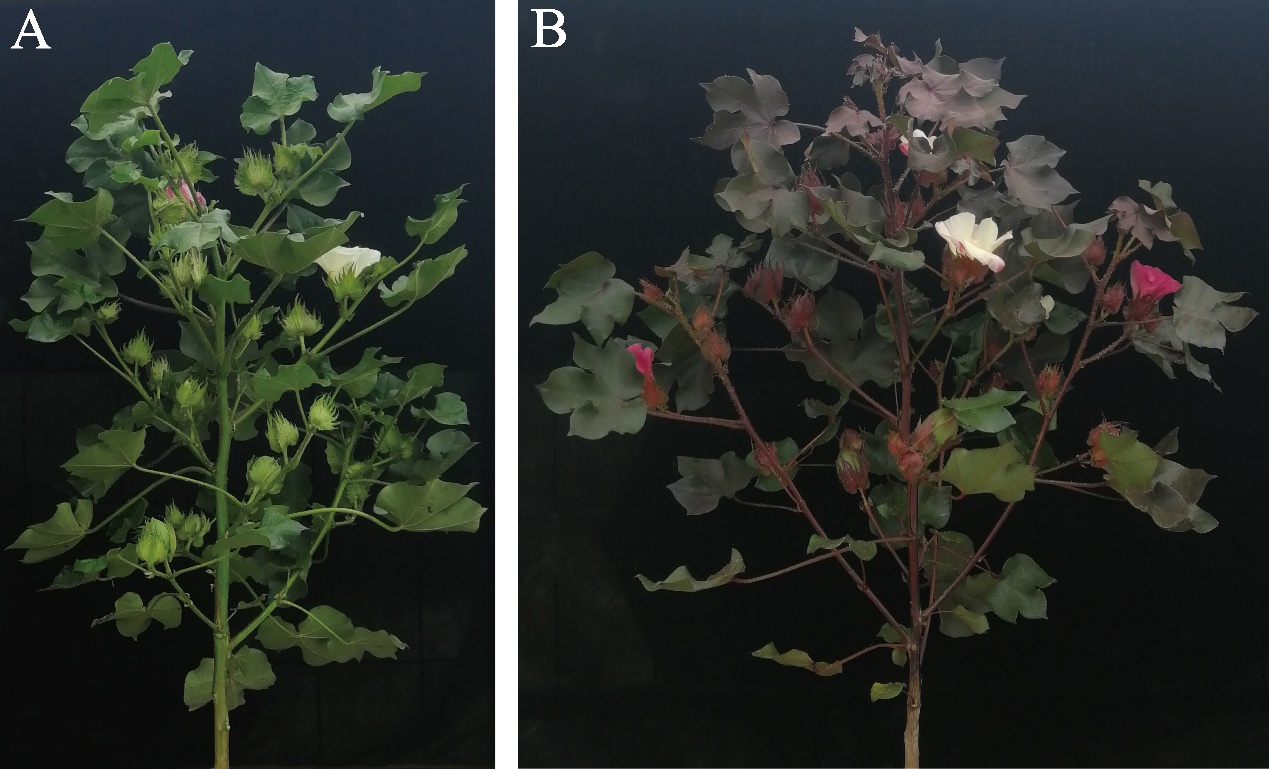
**

**Supplementary Figure S1.** Phenotypic comparison between (A) green leaf cotton XinLuZao 61 and (B) red leaf cottonHuiyuan cultivars grown in natural light.

**
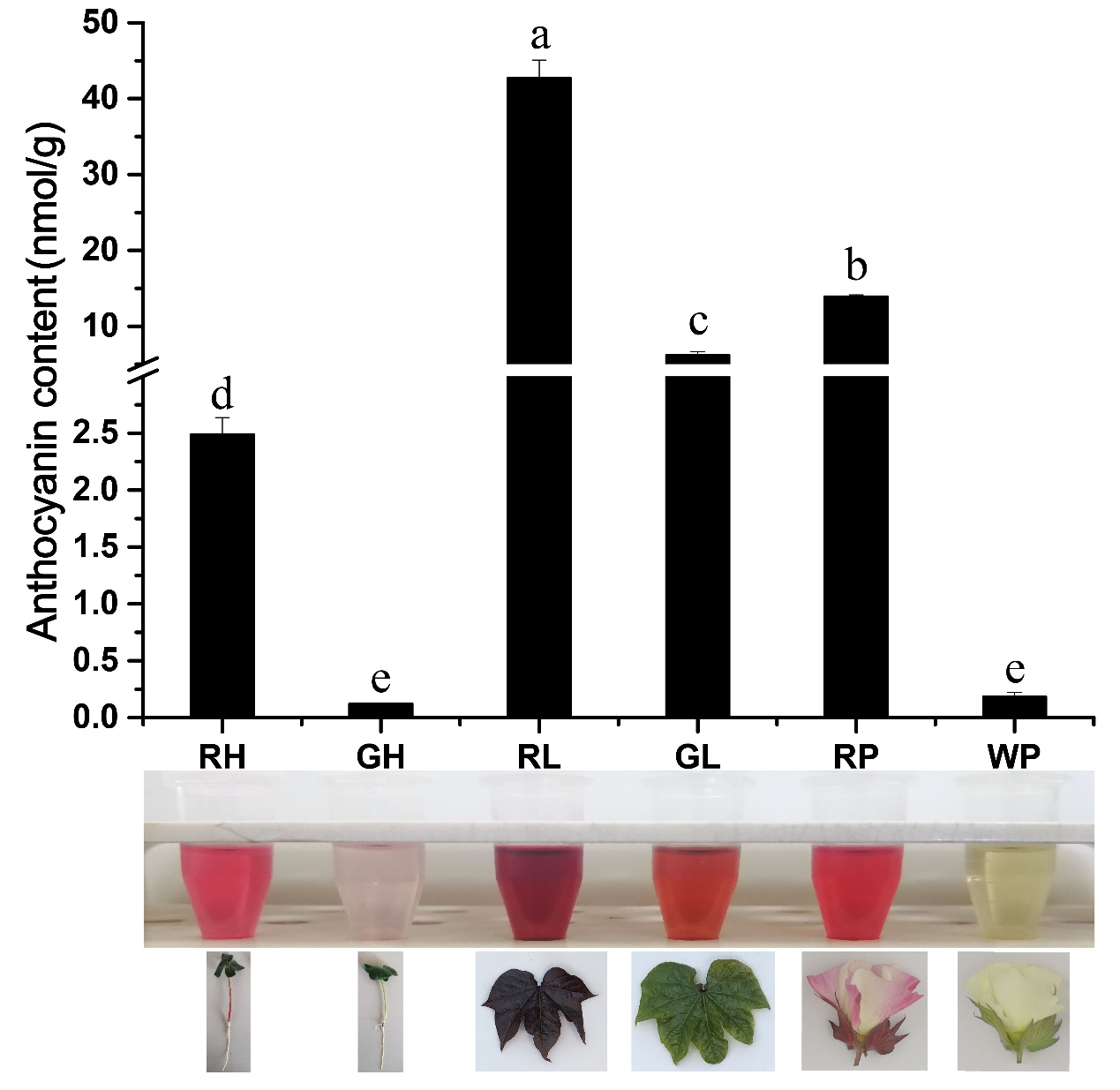
**

**Supplementary Figure S2.** Analysis of total anthocyanin concentrations of different tissues and organs from X61 (XinLuZao 61) and Huiyuan cultivars grown in light conditions. RH: Hypocotyl of Huiyuan; GH: Hypocotyl of X61; RL: Leaves of Huiyuan; GL: Leaves of X61; RP: Petals of Huiyuan; WP: Petals of X61. Three biological replicates of each sample were analyzed. Data are expressed as the means ± SD, n = 3. The letters on top of the bars indicate significance with the same letters being insignificant according to one-way analysis of variance (ANOVA) (P < 0.05).

**
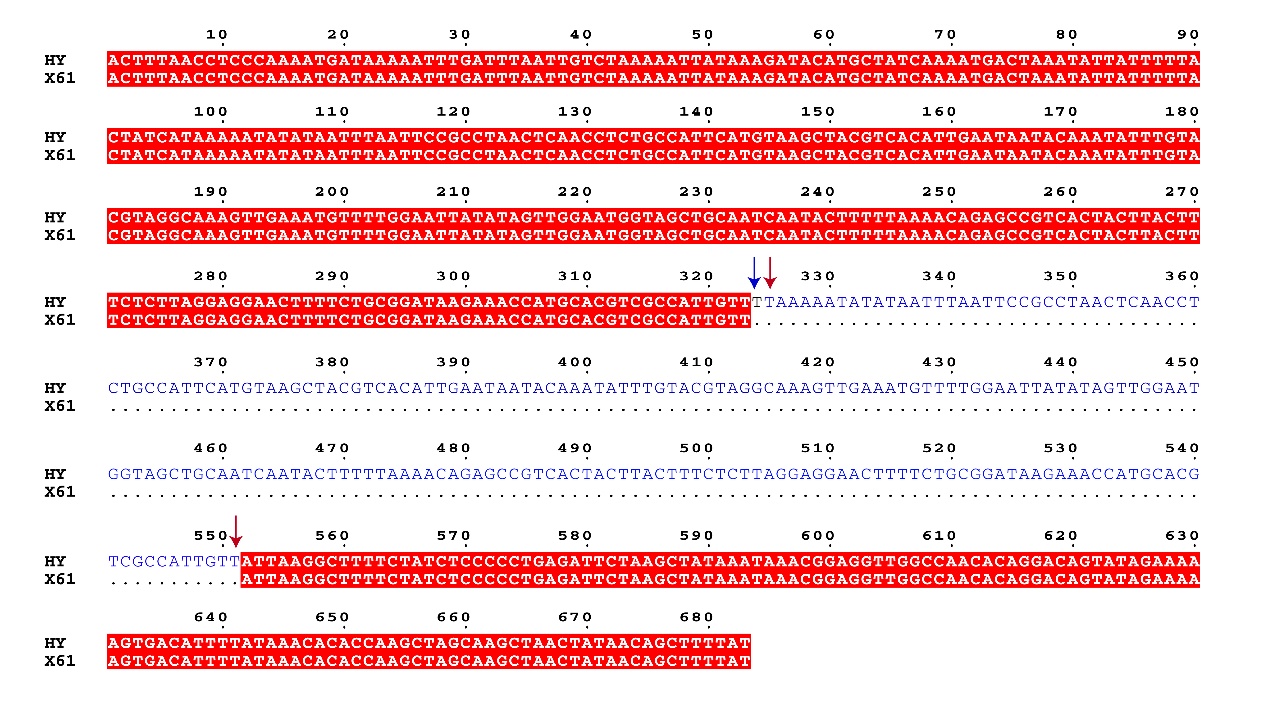
**

**Supplementary Figure S3.** Promoter alignment of *GhPAP1D* from XinLuZao 61 (X61) and Huiyuan (HY). The blue font indicates repeated bases. The blue arrow indicates the SNP between Huiyuan and *R1* promoter. The sequence between the two red arrows indicates a repeating region.

**
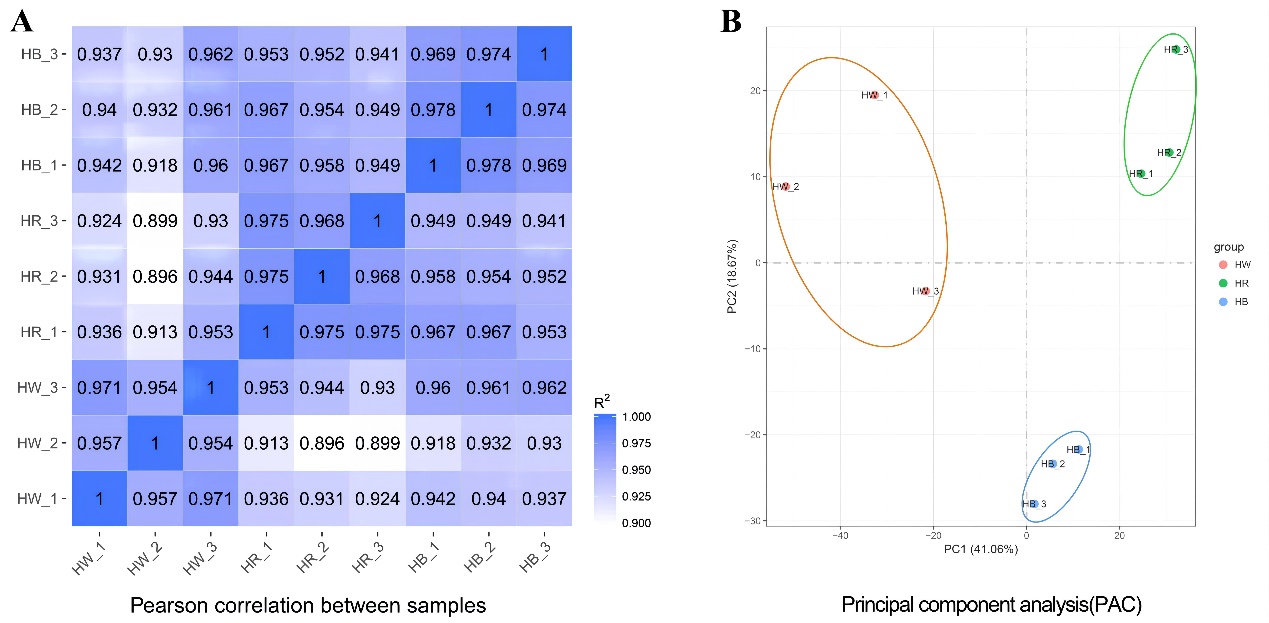
**

**Supplementary Figure S4.** Pearson correlation (A) and principal component analysis (B) between samples. HB, HR, and HW indicates samples treated by blue light, red light, and white light, respectively. HB_1, HB_2, HB_3; HR_1, HR_2, HR_3; HW_1, HW_2, HW_3; represents three biological replicates for each treatment, respectively.

**
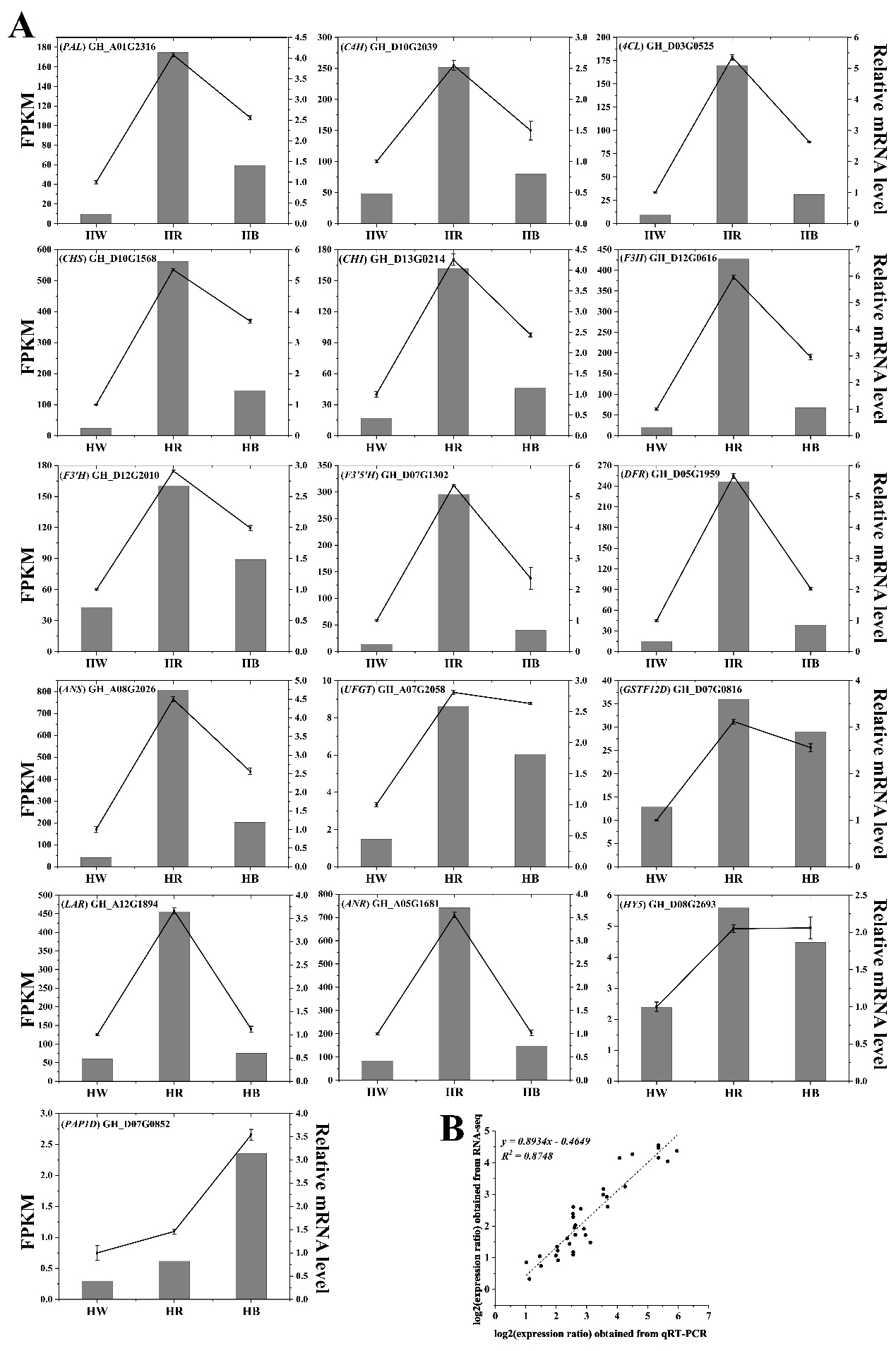
**

**Supplementary Figure S5.** Expression analysis of 16 differentially expressed genes related to anthocyanin biosynthesis in Huiyuan treated with different light quality. A: Transcript levels and qRT-PCR results of 16 selected genes from RNA-sequencing. The left y-axis shows the corresponding RNA-Seq expression data and the right y-axis indicates the relative gene expression levels analyzed by qRT-PCR. Each value in the histogram represents the mean ± standard error. B: The scatter plot of 16 selected genes based on the log2 of the gene expression ratios from qRT-PCR and RNA-seq results indicates the correlation between them. HW, HR, and HB indicates samples treated by white light, red light and blue light, respectively.

**
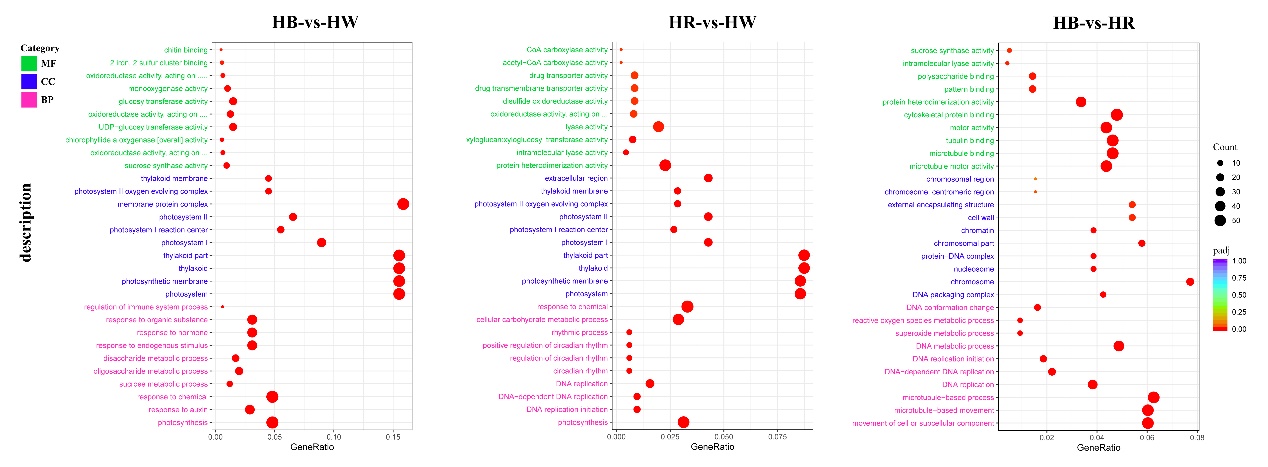
**

**Supplementary Figure S6.** Top 10 enriched Gene ontology (GO) classifications among the annotated DEGs in three pairwise comparisons (HB-vs-HW (left); HR-vs-HW (middle); and HB-vs-HR (right)). MF, CC, and BP represent molecular function, cellular component, and biological process, respectively. The Y-axis on the left represents GO entry names, and the X-axis indicates the enrichment gene ratio. Low q-values are shown in red, and high q-values are depicted in blue. Pathways with q-values less than 0.05 are significantly enriched. The size of the spot reflects the number of DEGs, and the color of the spot corresponds to different q-value ranges. HW, HR, and HB indicates samples treated by white light, red light, and blue light, respectively.

**
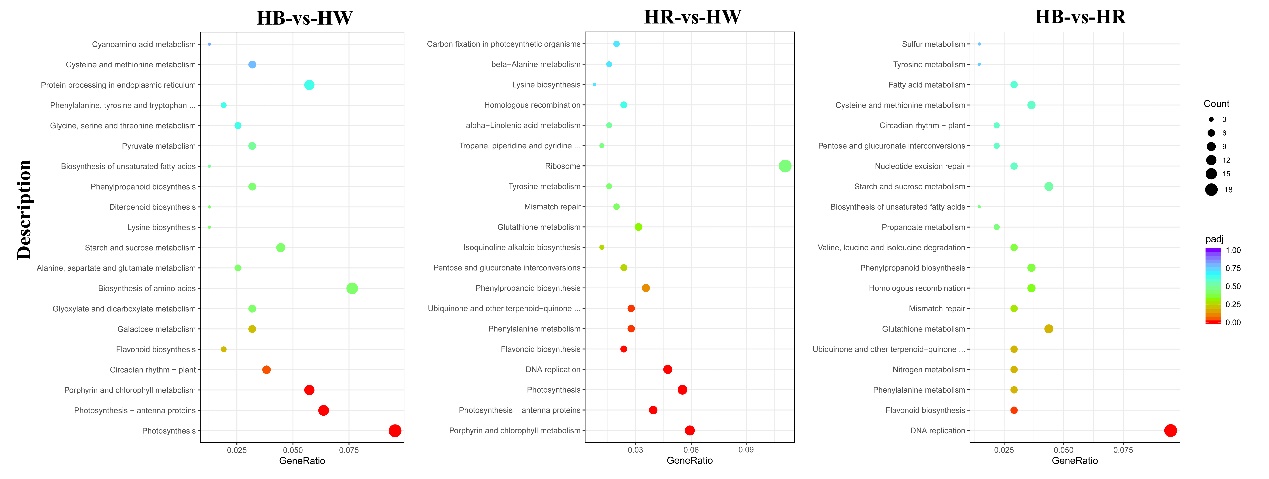
**

**Supplementary Figure S7.** Top 20 enriched KEGG pathways among the annotated DEGs in three pairwise comparisons (HB-vs-HW (left); HR-vs-HW (middle); and HB-vs-HR (right)). The Y-axis on the left represents KEGG pathways, and the X-axis indicates the enrichment gene ratio. Low q-values are shown in red, and high q-values are depicted in blue. Pathways with q-values less than 0.05 are significantly enriched. The size of the spot reflects the number of DEGs, and the color of the spot corresponds to different q-value ranges. HW, HR, and HB indicates samples treated by white light, red light, and blue light, respectively.

**
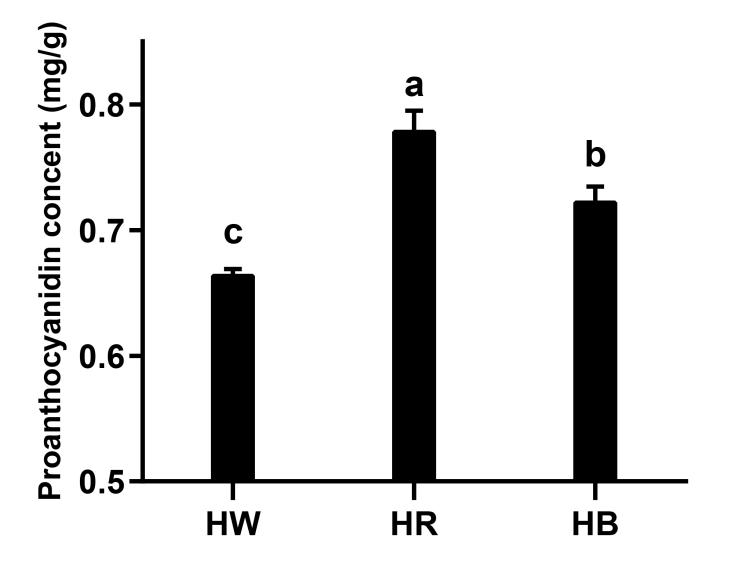
**

**Supplementary Figure S8.** The content of proanthocyanidins in Huiyuan leaves after different light quality treatment. Different lower-case letters indicate signiﬁcant differences based on one-way analysis by the Duncan test (P < 0.05). HW, HR, and HB indicates samples treated by white light, red light, and blue light, respectively. Three biological replicates of each sample were analyzed. Data are expressed as the means ± SD, n = 3.

**
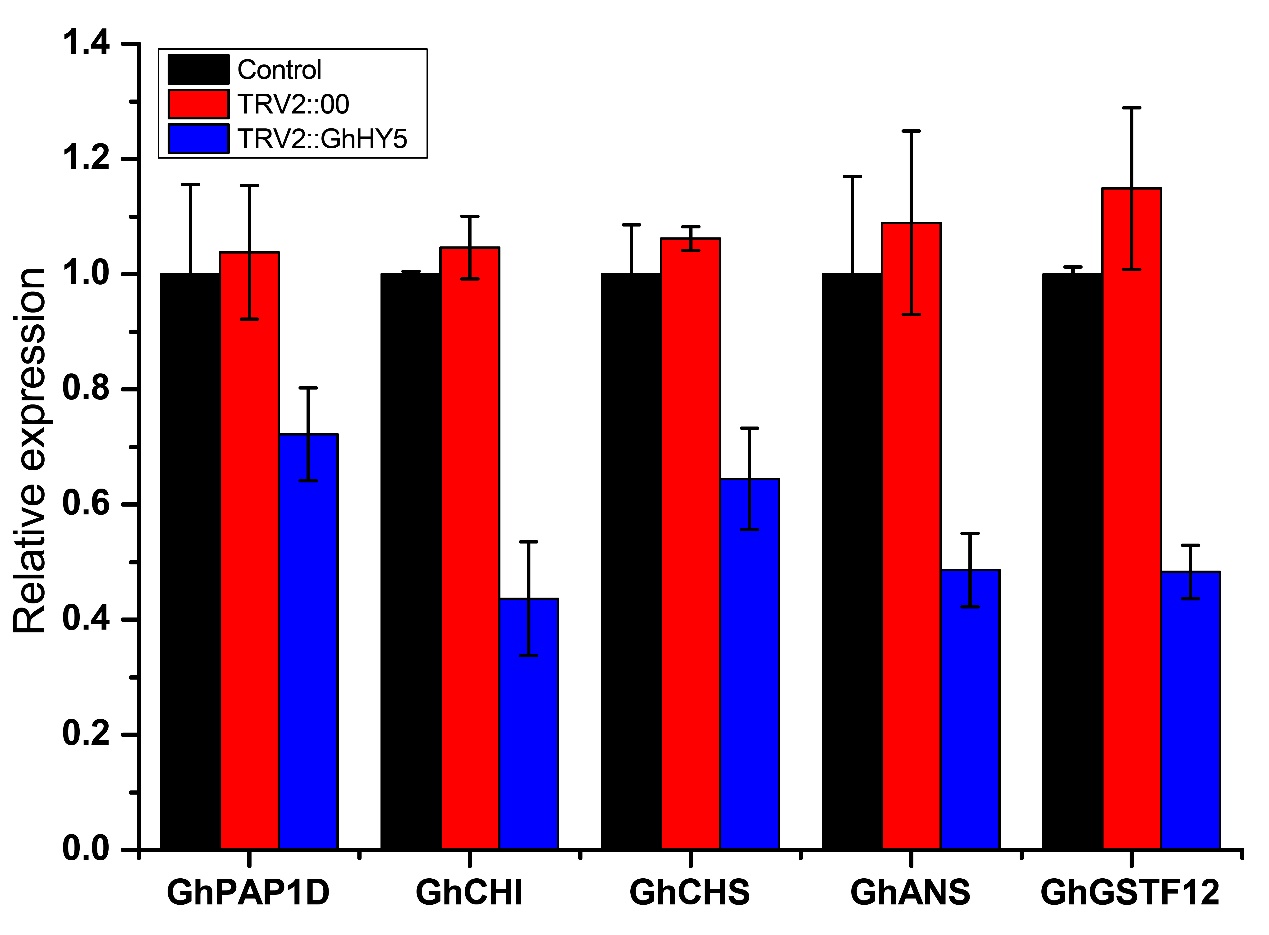
**

**Supplementary Figure S9.** Relative expression of genes related to anthocyanin biosynthesis pathway of Control, *TRV2::00*, and *TRV2::GhHY5*. GhPAP1D: MYB transcription factor of Upland Cotton; GhCHI: chalcone isomerase of Upland Cotton; GhCHS: chalcone synthase of Upland Cotton; GhANS: anthocyanidin synthase of Upland Cotton; GhANS: glutathione S-transferase F12 of Upland Cotton. Three biological replicates of each sample were analyzed. *GhUBQ7* was used as a housekeeping gene. Three biological replicates of each sample were analyzed. Data are expressed as the means ± SD, n = 3.
